# Meta-Analysis of Interleukin-2 Receptor Antagonists as the Treatment for Steroid-Refractory Acute Graft-*Versus*-Host Disease

**DOI:** 10.3389/fimmu.2021.749266

**Published:** 2021-09-21

**Authors:** Meng-Zhu Shen, Jing-Xia Li, Xiao-Hui Zhang, Lan-Ping Xu, Yu Wang, Kai-Yan Liu, Xiao-Jun Huang, Shen-Da Hong, Xiao-Dong Mo

**Affiliations:** ^1^Peking University People’s Hospital, Peking University Institute of Hematology, National Clinical Research Center for Hematologic Disease, Beijing Key Laboratory of Hematopoietic Stem Cell Transplantation, Beijing, China; ^2^Department of Hematology, The First Affiliated Hospital, Sun Yat-sen University, Guangzhou, China; ^3^Research Unit of Key Technique for Diagnosis and Treatments of Hematologic Malignancies, Chinese Academy of Medical Sciences, Beijing, China; ^4^Peking-Tsinghua Center for Life Sciences, Beijing, China; ^5^National Institute of Health Data Science at Peking University, Peking University Health Science Center, Beijing, China

**Keywords:** acute graft-versus-host disease, interleukin-2 receptor antagonist, second-line treatment, steroid-refractory, meta-analysis

## Abstract

Acute graft-versus-host disease (aGVHD) is a major complication after allogeneic hematopoietic stem cell transplantation (HSCT). Corticosteroid is the first-line treatment for aGVHD, but its response rate is only approximately 50%. At present, no uniformly accepted treatment for steroid-refractory aGVHD (SR-aGVHD) is available. Blocking interleukin-2 receptors (IL-2Rs) on donor T cells using pharmaceutical antagonists alleviates SR-aGVHD. This meta-analysis aimed to compare the efficacy and safety of four commercially available IL-2R antagonists (IL-2RAs) in SR-aGVHD treatment. A total of 31 studies met the following inclusion criteria (1): patients of any race, any sex, and all ages (2); those diagnosed with SR-aGVHD after HSCT; and (3) those using IL-2RA-based therapy as the treatment for SR-aGVHD. The overall response rate (ORR) at any time after treatment with basiliximab and daclizumab was 0.81 [95% confidence interval (CI): 0.74–0.87)] and 0.71 (95% CI: 0.56–0.82), respectively, which was better than that of inolimomab 0.54 (95% CI: 0.39–0.68) and denileukin diftitox 0.56 (95% CI: 0.35–0.76). The complete response rate (CRR) at any time after treatment with basiliximab and daclizumab was 0.55 (95% CI: 0.42–0.68) and 0.42 (95%CI: 0.29–0.56), respectively, which was better than that of inolimomab 0.30 (95% CI: 0.16–0.51) and denileukin diftitox 0.37 (95% CI: 0.24–0.52). The ORR and CRR were better after 1-month treatment with basiliximab and daclizumab than after treatment with inolimomab and denileukin diftitox. The incidence of the infection was higher after inolimomab treatment than after treatment with the other IL-2RAs. In conclusion, the efficacy and safety of different IL-2RAs varied. The response rate of basiliximab was the highest, followed by that of daclizumab. Prospective, randomized controlled trials are needed to compare the efficacy and safety of different IL-2RAs.

## Introduction

Hematopoietic stem cell transplantation (HSCT) is a curative measure for hematopoietic malignancies (1). However, its outcome has been compromised by acute graft-versus-host disease (aGVHD), which is a major complication that occurs early post-HSCT. Although many efforts have been made to prevent aGVHD, it is still responsible for early mortality post-transplantation (2). Corticosteroid is the first-line treatment of aGVHD. However, its response rate is only approximately 50% (3). Thus far, no universally accepted treatment for steroid-refractory aGVHD (SR-aGVHD) is available, and survival is poor (4).

One of the critical pathophysiological mechanisms of aGVHD is mediated by T-lymphocyte activation, which exclusively expresses the interleukin-2 receptor (IL-2R) alpha chain (5). Blocking IL-2R on donor T cells using pharmaceutical antagonists alleviates aGVHD, especially SR-aGVHD (6). Some commercially available IL-2R antagonists (IL-2RAs) are basiliximab, daclizumab, inolimomab, and denileukin diftitox. The first three are monoclonal antibodies, which can directly interrupt subsequent T-cell activation by binding to CD25 with high affinity. Inolimomab is a murine anti-human monoclonal antibody with a half-life of 44.5 h (7). Basiliximab is a murine chimeric monoclonal antibody with a half-life of 7 days (8). Daclizumab is a humanized monoclonal antibody with a half-life of 21–25 days (9). In addition, denileukin difititox is a recombinant fusion protein made of diphtheria toxin and human IL-2 sequence, which binds to IL-2R and poisons activated T lymphocytes afterward (10). The half-life of denileukin difititox is 70–80 min (11). Since the 1990s, emerging studies have identified the efficacy and safety of these IL-2RAs in SR-aGVHD treatment (8, 10, 12–41); however, the results varied dramatically because of the great heterogeneity in the study design. So far, no study has been designed to compare the efficacy of different IL-2RAs.Thus, this meta-analysis was conducted to compare the efficacy and safety of these four IL-2RAs in SR-aGVHD treatment.

## Methods

### Inclusion Criteria

The inclusion criteria were as follows (1): patients of any race, any sex, and all ages (2); those diagnosed with SR-aGVHD after HSCT; and (3) those using IL-2RA-based therapy as the treatment for SR-aGVHD. Reviews, case reports, duplicates, and conference abstracts were excluded. Multiple studies reporting the same data were considered as one.

### Search Strategy

A literature search was conducted following the Preferred Reporting Items for Systematic Reviews and Meta-analyses statement (42). The PubMed and Embase databases were searched, published from January 2000 through December 2020, with the search strategy following the Population (patients with steroid refractory acute graft versus host disease), Intervention (interleukin-2 receptor antagonists), Comparison(between four different interleukin-2 receptor antagonists), Outcomes (overall response rate [ORR], complete response rate [CRR], chronic GVHD [cGVHD], overall survival [OS] rate, and infectious complications), and Study framework (retrospective, prospective non-randomized and randomized trials) (43): (interleukin-2 OR IL-2 OR CD25 OR Daclizumab OR Basiliximab OR Inolimomab OR Denileukin) AND (steroid refractory OR steroid-refractory OR steroid resistant OR steroid-resistant OR corticosteroid refractory OR corticosteroid-refractory) AND (acute graft versus host disease OR aGVHD) AND 2000/01/01[dp]:2020/12/31[dp].

### Data Extraction and Outcomes

The ORR, CRR, cGVHD, overall survival rate, and infectious complications at any time after treatment with IL-2RAs were chosen as the primary end points. In addition, the response rate at 1 month after IL-2RA treatment was assessed. As different studies had different time points, the time frame for the evaluation of response rate at 1 month after IL-2RA treatment was prolonged. That is, the earliest studies evaluating at 3 weeks while the latest studies evaluating at 6 weeks after treatment with IL-2RAs were enrolled in this analysis. Missing data were documented as “not available (NA)”.

### Statistical Analysis

The “meta” package version 4.18-0 (44) (R Project for Statistical Computing, version 4.0.5) was used to perform the meta-analysis. Statistical heterogeneity among studies was assessed using the *I* ^2^ statistics and Cochran Q-test. The random-effects model was adopted, with the heterogeneity test showing *I* ^2^ > 50% and *P* < 0.10. Also, the “stats” package version 4.0.5 (45) was used to perform the *t* test for comparison between the means of two subgroups, including the organ response rates, infection rates, cGVHD rates and OS rates. The null hypothesis was set to no difference. A *P* value <0.05 was considered statistically significant to reject the null hypothesis.

## Results

### Included Studies

**Figure 1:**
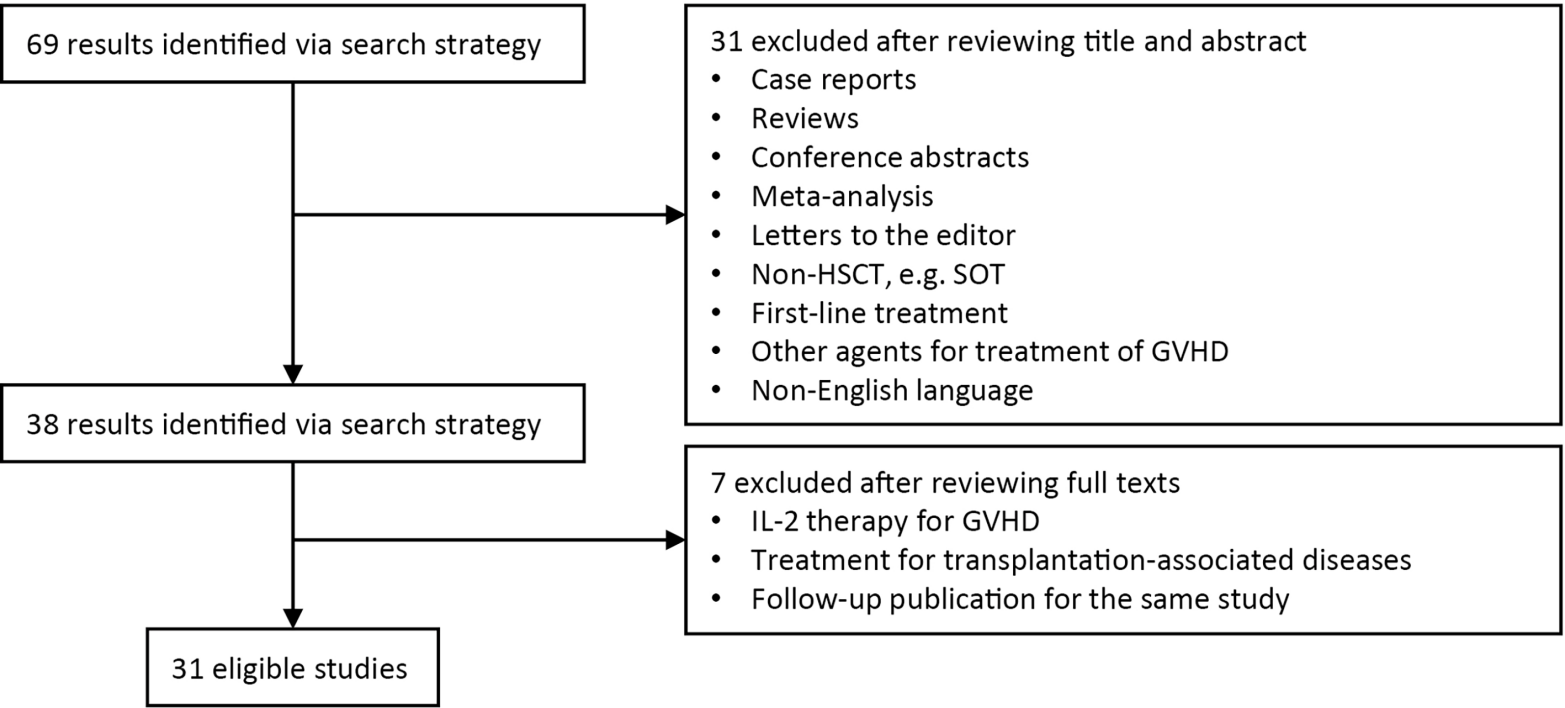
Selection scheme of studies. GVHD, graft-versus-host disease; HSCT, hematopoietic stem cell transplant; SOT, solid organ transplant.

A total of 31 studies reporting on basiliximab (8, 13–20), daclizumab [(22–32); one study using the domestic generic drug (21)], inolimomab (33–40), and denileukin diftitox (10, 41) were included in this meta-analysis (**Figure 1**). A total of 1360 patients were enrolled, including 533, 337, 438, and 52 patients treated with basiliximab, daclizumab, inolimomab, and denileukin diftitox, respectively (**Tables 1**, **2**, and **Supplementary Table 1**). Three (15–17), four (22, 23, 29, 30), and two (34, 36) studies used combined therapies of basiliximab, daclizumab, and inolimomab, respectively.

**Table 1:** Main characteristics of 31 included studies.

Studies	Study design	N	Response/event (n)				cGVHD incidence (%)	Overall survival rate	median follow-up (months)
			ORR	ORR at 1 month	CR	CR at 1 month			
**Basiliximab-based treatment**									
Liu, (13)	retrospective	230	151	151	140	140	44.80	0.617	41.8
Tang, (14)	retrospective	100	85	85	74	74	43.75	0.762	25.3
Tan, (15)	prospective, unrandomized	65	59	59	49	49	50.00	0.547	18.5
Chakupurakal, (8)	prospective, unrandomized	14	13	NA	7	NA	NA	NA	NA
Nadeau, (16)	retrospective	21	16	16	9	9	71.43	0.24	34.5
Jaiswal, (17)	prospective, unrandomized	10	7	7	3	3	16.67	NA	4.2
Wang, (18)	retrospective	53	46	NA	37	NA	69.39	0.514	0.2
Schmidt-Hieber, (19)	prospective, unrandomized	23	19	NA	4	NA	62.50	NA	6.1
Massenkeil, (20)	retrospective	17	12	NA	9	NA	61.54	NA	4.1
**Daclizumab-based treatment**									
Tao, (21)	retrospective	64	53	53	37	37	34.38	0.729	0.1
Rager, (22)	retrospective	17	8	NA	4	NA	NA	NA	1.5
Rao, (23)	retrospective	22	19	NA	12	NA	50.00	NA	16.2
Miano, (24)	retrospective	13	12	12	6	6	66.67	NA	14.0
Hui, (25)	retrospective	12	2	2	1	1	NA	NA	4.0
Perales, (26)	retrospective	57	31	31	NA	NA	NA	NA	98.0
Teachey, (27)	retrospective	11	7	NA	5	NA	NA	NA	NA
Bordigoni, (28)	prospective, unrandomized	62	56	56	43	43	67.80	NA	1.5
Wolff, (29)	prospective, unrandomized	21*	14	NA	8	NA	66.67	NA	19.5
Srinivansan, (30)	retrospective	3	3	NA	3	NA	100.00	NA	4.0
Willenbacher, (31)	prospective, unrandomized	12	8	8	1	1	NA	NA	15.3
Preziprka, (32)	prospective, unrandomized	43	22	22	16	16	NA	0.4	2.6
**Inolimomab-based treatment**									
Girerd, (34)	prospective, randomized	49	NA	NA	NA	NA	NA	0.469	58.4
Garcia-Cadenas, (35)	retrospective	98	38	38	NA	NA	NA	0.454	19.4
van Groningen, (36)	prospective, unrandomized	21	10	10	6	6	NA	0.1	1.8
Girerd, (37)	retrospective	33	30	30	24	24	63.33	0.79	NA
Garcia-Cadenas, (35)	retrospective	92	39	39	13	13	NA	0.22	60.0
Xhaard, (38)	retrospective	20	7	NA	NA	NA	NA	0.3	74.0
Pinana, (39)	retrospective	40	20	20	8	8	78.26	0.3	1.9
Bay, (40)	retrospective	85	54	NA	25	NA	NA	0.26	20.0
**Denileukin diftitox treatment**									
Shaughnessy, (10)	prospective, unrandomized	22	9	9	9	9	60.00	NA	4.0
Ho, (41)	prospective, unrandomized	30	17	17	8	8	NA	NA	7.2

NA, not available; ORR, overall response rate; CR, complete response rate; cGVHD, chronic graft-versus-host disease.

*Only 20 patients were eligible for evaluation because 1 patient died 5 days after treatment due to rapid progression of pre-existing invasive aspergillosis.

**Table 2:** Other characteristics of 31 included studies.

Studies	Median age/year (range)	HLA matching (*n*)				aGVHD grade (*n*)				Median time from SR-aGVHD diagnosis to the application of IL-2RAs/day (range)
		MRD	mMRD	MUD	mMUD	I	II	III	IV	
**Basiliximab-based treatment**										
Liu, (13)	NA	17	208	5		0	191	25	14	5 (3–20)
Tang, (14)	10 (1–17)	NA	NA	NA	NA	0	57	27	16	NA
Tan, (15)	13 (9–55)	13	40	12	0	0	0	21	44	8 (3–49)
Chakupurakal, (8)	41 (20–69)	0	1	6	7	1	1	5	7	NA
Nadeau, (16)	57 (20–71)	7	1	10	3	0	0	13	8	5(NA)
Jaiswal, (17)	7 (2–20)	0	10	0	0	0	0	10		NA
Wang, (18)	25 (8–52)	NA	NA	NA	NA	0	10	27	16	NA
Schmidt-Hieber, (19)	51 (31–63)	7	1	12	3	0	11	12	0	NA
Massenkeil, (20)	39 (23–50)	6	0	11	0	0	3	12	2	7 (3–25)
**Daclizumab-based treatment**										
Tao, (21)	35 (13–57)		45		19	0	3	28	33	NA
Rager, (22)	47 (35–63)	5	0	9	2	0	3	10	4	7 (2–26)
Rao, (23)	NA	4	0	12	6	0	0	7	15	NA
Miano, (24)	NA	3		10		0	4	4	5	48 (12–201)
Hui, (25)	38.5 (25–55)	9	0	2	1	0	0	12	0	8.5 (3–28)
Perales, (26)	28.9 (0.7–57.7)	21	12	13	11	5	23	14	15	NA
Teachey, (27)	NA	NA	NA	NA	NA	0	6	3	2	NA
Bordigoni, (28)	25.4 (1.5–53)	32	1	11	18	0	41	21		NA
Wolff, (29)	44 (15–61)	6	0	14	1	0	1	17	3	17 (3–66)
Srinivansan, (30)	33 (33–46)	NA	NA	NA	NA	0	0	1	2	NA
Willenbacher, (31)	46 (28–56)	3	1	5	3	0	0	1	11	5 (3–13)
Preziprka, (32)	31 (1–53)	14	15	14	0	1	22	12	8	NA
**Inolimomab-based treatment**										
Girerd, (34)	NA	15	0	31	3	0	0	100	0	6 (3–9)
Garcia-Cadenas, (36)	50 (17–70)	54	44			0	6	51	41	15 (4–91)
van Groningen, (36)	54 (24–66)	11	0	7	3	0	0	17	4	NA
Girerd, (34)	44 (17–65)	6	9	11	7	0	7	19	7	15 (3–36)
Garcia-Cadenas, (35)	50 (17–68)	52	40			0	66	48	38	17 (2–204)
Xhaard, (38)	42 (5–64)	4	0	11	5	0	13	7	0	12 (NA)
Pinana, (39)	47 (17–63)	27	1	5	7	0	2	22	16	21 (4–91)
Bay, (40)	29.5 (0.2–61)	41	8	27	9	0	26	26	33	13 (8–23)
**Denileukin diftitox treatment**										
Shaughnessy, (10)	44 (9–59)	12	0	8	2	0	7	7	8	NA
Ho, (41)	43 (20–63)	2	26	1	1	0	11	13	6	NA

NA, not available; HLA, human leukocyte antigen; MRD, matched related donor; MUD, matched unrelated donor; mMRD, mismatched related donor; mMUD, mismatched unrelated donor; aGVHD, acute graft-versus-host disease; IL-2RAs, interleukin-2 receptor antagonists.

### ORR After Treatment With IL-2RAs

**Figure 2:**
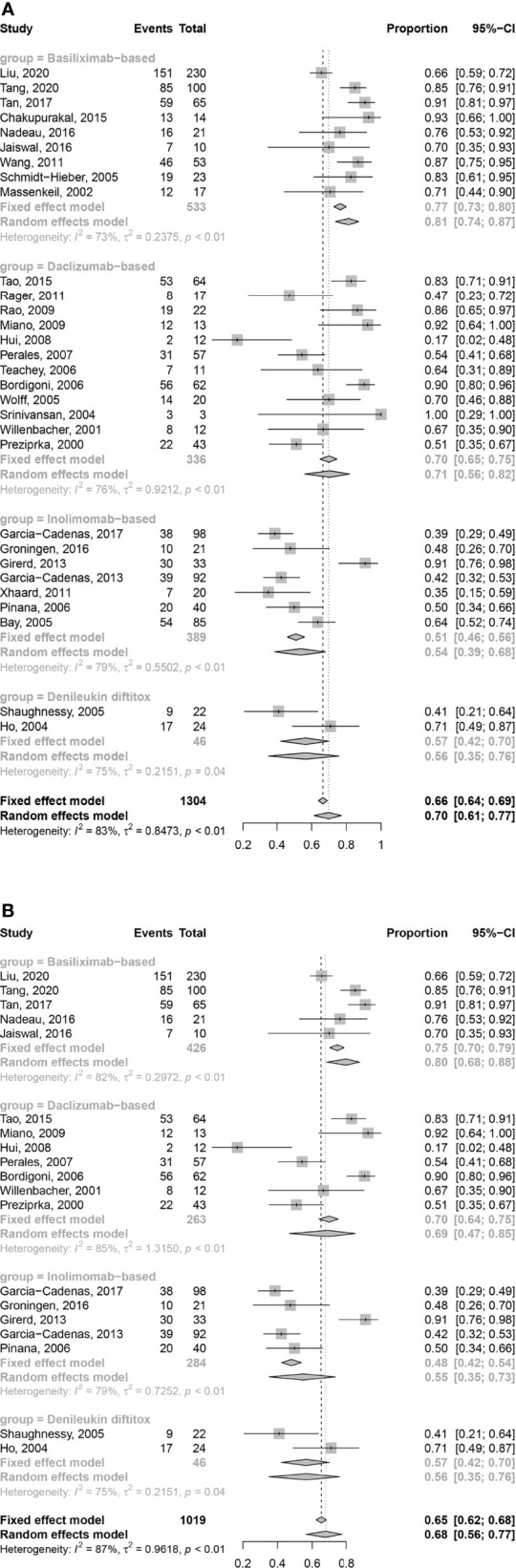
Forest plots of ORR at any time **(A)** and 1 month **(B)** after treatment with IL-2RAs.

The results showed that ORR of basiliximab and daclizumab was 0.81 [95% confidence interval (CI): 0.74–0.87] and 0.71 (95% CI: 0.56–0.82), respectively, which seemed to be better than that of inolimomab 0.54 (95% CI: 0.39–0.68) and denileukin diftitox 0.56 (95% CI: 0.35–0.76) (**Figure 2A**).

In retrospective studies, ORR of basiliximab, daclizumab and inolimomab was 0.78 (95% CI: 0.68–0.85), 0.70 (95% CI: 0.48–0.85) and 0.55 (95% CI: 0.37–0.71), respectively. In prospective unrandomized studies, ORR of basiliximab, daclizumab, inolimomab, and denileukin diftitox was 0.87 (95% CI: 0.80–0.92), 0.73 (95% CI: 0.53–0.86), 0.48 (95% CI: 0.28–0.68), and 0.56 (95% CI: 0.35–0.76), respectively (**Supplementary Figure S1**). Only 1 randomized controlled trial (RCT) identifying the efficacy of inolimomab about SR-aGVHD did not provide the data about ORR (33).

In the analysis of ORR at 1 month after treatment, 4 (8, 18–20), 5 (22, 23, 27, 29, 30), and 2 (38, 40) studies on basiliximab, daclizumab, and inolimomab were excluded due to insufficient data. The ORR at 1 month after treatment with IL-2RA of basiliximab and daclizumab was 0.80 (95% CI: 0.68–0.88) and 0.69 (95% CI: 0.47–0.85), respectively, which seemed to be better than that of inolimomab 0.55 (95% CI: 0.35–0.73) and denileukin diftitox 0.56 (95% CI: 0.35–0.76) (**Figure 2B**).

In retrospective studies, the ORR at 1 month after treatment with IL-2RA of basiliximab, daclizumab, and inolimomab was 0.76 (95% CI: 0.63–0.85), 0.66 (95% CI: 0.31–0.89), and 0.57 (95% CI: 0.33–0.79), respectively. In prospective unrandomized studies, the ORR at 1 month after treatment with IL-2RA of basiliximab, daclizumab, inolimomab, and denileukin diftitox was 0.88(95% CI: 0.79-0.94), 0.73 (95% CI: 0.47–0.89), 0.48 (95% CI: 0.28–0.68), and 0.56 (95% CI: 0.35–0.76), respectively (**Supplementary Figure S2**). The RCT about inolimomab did not provide the data about ORR at 1 month after treatment (33).

### CRR After Treatment With IL-2RAs

**Figure 3:**
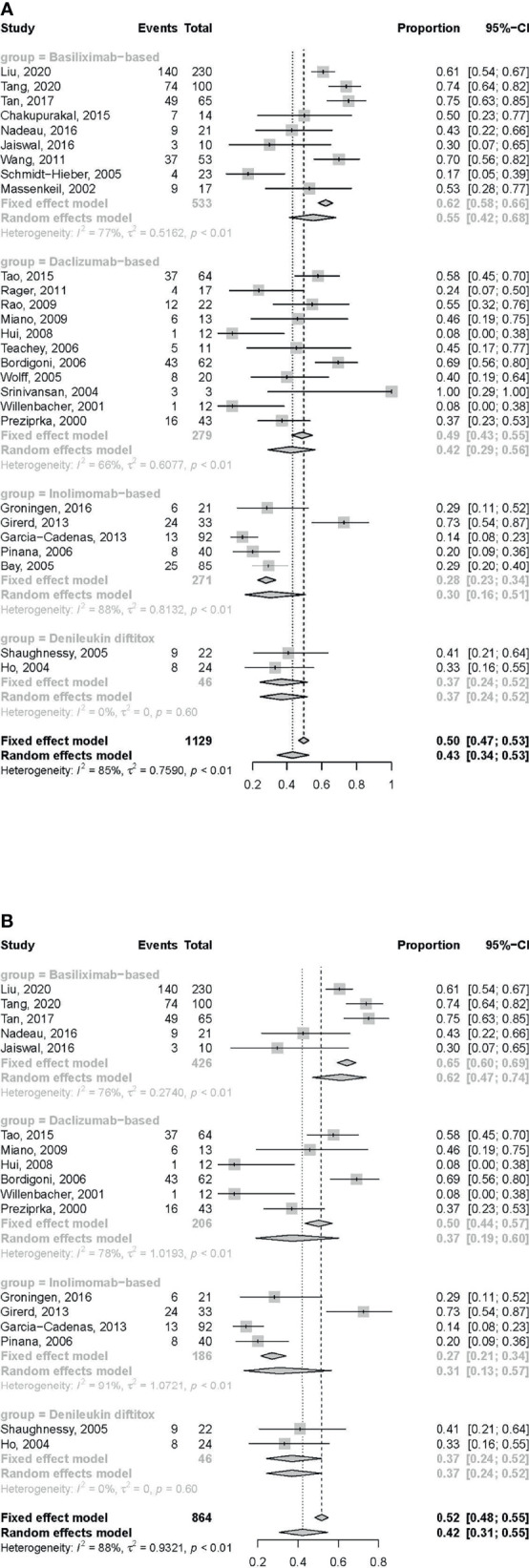
Forest plots of CRR at any time **(A)** and 1 month **(B)** after treatment with IL-2RAs.

The CRR at any time after treatment with basiliximab and daclizumab was found to be 0.55 (95% CI: 0.42–0.68) and 0.42 (95%CI: 0.29–0.56), respectively, which was better than that of inolimomab 0.30 (95% CI: 0.16–0.51) and denileukin diftitox 0.37 (95% CI: 0.24–0.52) (**Figure 3A**).

In retrospective studies, the CRR of basiliximab, daclizumab and inolimomab was 0.64 (95% CI: 0.55–0.72), 0.44 (95% CI: 0.29–0.60), 0.31 (95% CI: 0.14–0.56), respectively. In prospective unrandomized studies, CRR of basiliximab, daclizumab, inolimomab and denileukin diftitox was 0.44 (95% CI: 0.21–0.70), 0.39 (95% CI: 0.19–0.64), 0.29 (95% CI: 0.13–0.51), and 0.37 (95% CI: 0.24–0.52), respectively (**Supplementary Figure S3**). The RCT about inolimomab did not provide the data about CRR (33).

In the analysis of CRR at 1 month after treatment, 4 (8, 18–20), 5 (22, 23, 27, 29, 30), and 1 (40) studies on basiliximab, daclizumab, and inolimomab, respectively, were excluded due to insufficient data. The CRR at 1 month after treatment with IL-2RAs of basiliximab and daclizumab was found to be 0.62 (95% CI: 0.47–0.74) and 0.37 (95% CI: 0.19–0.60), respectively, which was better than that of inolimomab 0.31 (95% CI: 0.13–0.57) and denileukin diftitox 0.37 (95% CI: 0.24–0.52) (**Figure 3B**).

In retrospective studies, the CRR at 1 month after treatment with IL-2RAs of basiliximab, daclizumab and inolimomab was 0.63 (95% CI: 0.50–0.74), 0.37 (95% CI: 0.15–0.67) and 0.32 (95% CI: 0.10–0.66), respectively. In prospective unrandomized studies, the CRR at 1 month after treatment with IL-2RAs of basiliximab, daclizumab, inolimomab and denileukin diftitox was 0.58 (95% CI: 0.25–0.85), 0.38 (95% CI: 0.13–0.71), 0.29 (95% CI: 0.13–0.51) and 0.37 (95% CI: 0.24–0.52), respectively (**Supplementary Figure S4**). The RCT about inolimomab did not provide the data about CRR at 1 month after treatment (33).

### Response According to the Involved Organs After Treatment With IL-2RAs

The ORRs and CRRs of different organs involved for these four drugs were compared. Five (13–15, 19, 20), five (21, 24–26, 31), two (38, 40), and one (41) studies on basiliximab, daclizumab, inolimomab, and denileukin diftitox, respectively, were included in the analysis of the ORRs. The ORRs of skin, gut, and liver at any time after treatment were all comparable among these four IL-2RAs (**Supplementary Figure S5A**).

Two (15, 20), five (21, 24, 25, 31, 32), and two (10, 41) studies on basiliximab, daclizumab, and denileukin diftitox, respectively, were included in the analysis of CRRs. In the gut and liver aGVHD, basiliximab showed a higher CRR at any time compared with daclizumab [gut: 0.76 (95% CI: 0.34–1.19) *vs* 0.34 (95% CI: 0.05–0.62), *P* = 0.012; liver: 0.74 (95% CI: 0.67–0.82) *vs* 0.14 (95% CI: 0.08–0.20), *P* < 0.001; **Supplementary Figure S5B**].

### Infections After Treatment With IL-2RAs

Seven (13–15, 17–20), nine (21–26, 28, 29, 31), five (33–35, 37, 39), and two (10, 41) studies on basiliximab, daclizumab, inolimomab, and denileukin diftitox, respectively, were enrolled to analyze the incidence of infection after IL-2RA treatment. Two of them did not have information on viral infection and were excluded from the analysis of viral infections [daclizumab (25) and denileukin diftitox (41)]. The incidence of infection after inolimomab treatment [1.65 cases per person (95% CI: 0.78–2.53 cases per person)] was the highest compared with other IL-2RAs. The infection rates were comparable between the basiliximab group [1.19 cases per person (95% CI: 0.51–1.86 cases per person)] and the daclizumab group [0.95 cases per person (95% CI: 0.58–1.32 cases per person)], which both seemed to be higher than those in the denileukin diftitox group [0.24 cases per person (95% CI: 0–1.76 cases per person)]. The frequencies of viral infection were comparable among the four IL-2RAs (**Supplementary Figure S6**).

### cGVHD

Eight (13–20), six (21, 23, 24, 28–30), two (34, 39) and one (10) studies on basiliximab, daclizumab, inolimomab and denileukin diftitox, respectively, could be enrolled in the analysis of cGVHD. The incidence of cGVHD after basiliximab, daclizumab, inolimomab and denileukin diftitox treatment was 52.5% (95% CI: 37.5%–67.5%), 64.3% (95% CI: 41.3%–87.2%), 70.8% (95% CI: 24.0%–100.0%) and 60.0%, respectively. In retrospective studies, the incidence of basiliximab, daclizumab and inolimomab was 58.2% (95% CI: 41.8%–74.6%), 62.8% (95% CI: 18.0%–100.0%) and 70.8% (95% CI: 24.0%–100.0%), respectively. In prospective unrandomized studies, the incidence of basiliximab, daclizumab and denileukin diftitox was 43.1% (95% CI: 15.8%–100.0%), 67.2% (95% CI: 60.1%–74.4%) and 60.0%, respectively. The RCT about inolimomab did not provide the data about cGVHD (33).

### OS

**Figure 4:**
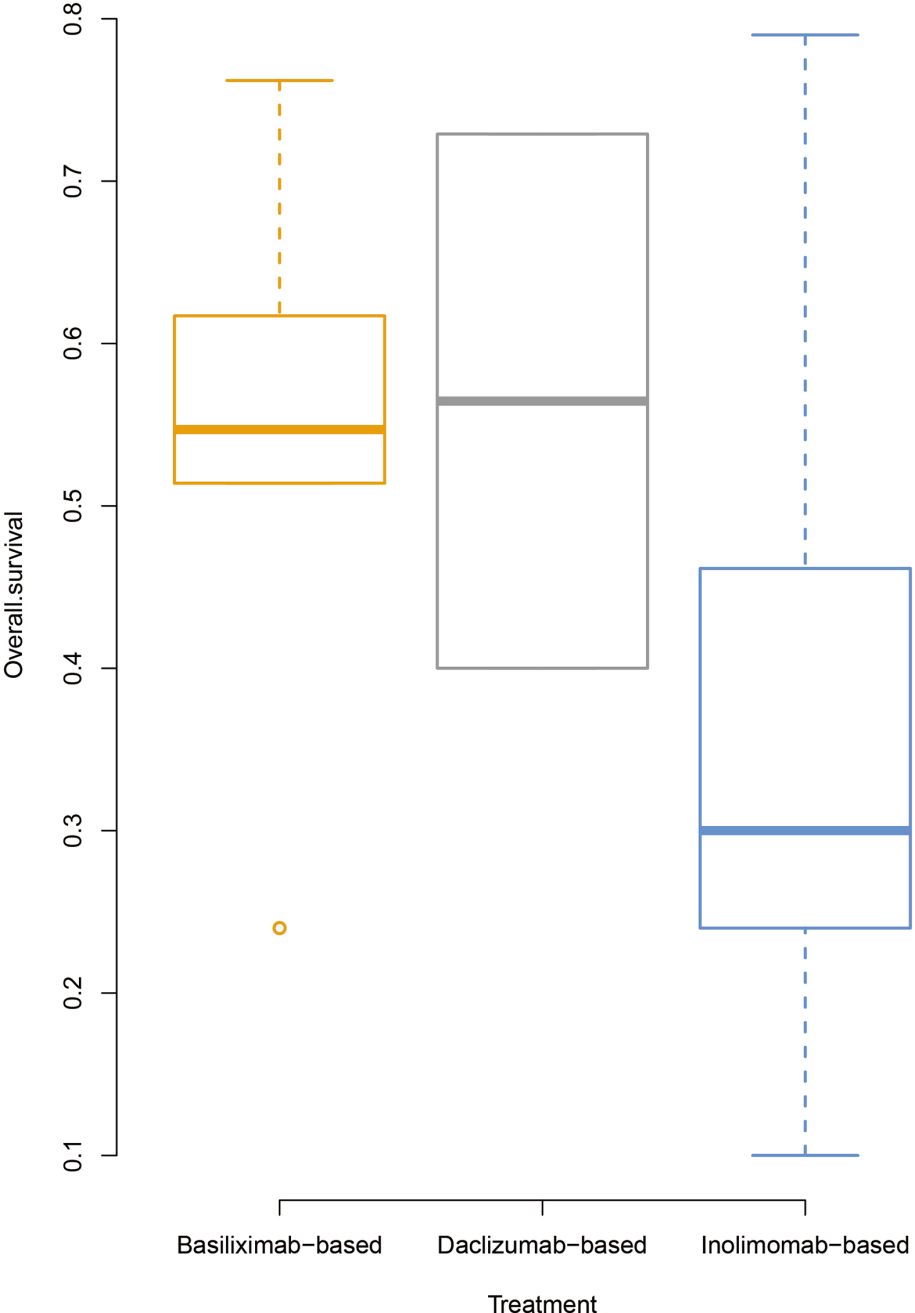
Overall survival rates of patients after treatment with IL-2RAs.

Five (13–16, 18), two (21, 32), and eight (33–40) studies on basiliximab, daclizumab, and inolimomab, respectively, were included in the survival analysis. Two studies on denileukin diftitox were excluded because they did not provide the information on OS. The OS rate for basiliximab and daclizumab was 53.6% (95% CI: 29.9%–77.31%) and 56.5% (only two studies were enrolled, ranging from 40% to 72.9%), respectively, which seemed to be higher than that in the inolimomab group [36.2% (95% CI: 18.6%–53.8%)] (**Figure 4**).

In retrospective studies, only one study for daclizumab could be observed and its OS rate was 72.9%. The OS rate of basiliximab and inolimomab was 53.3% (95% CI: 18.3%–88.4%) and 38.7% (95% CI: 16.4%–61.0%), respectively. One study each for basiliximab, daclizumab, and inolimomab could be included in the analysis of prospective unrandomized studies, and the OS rate was 54.7%, 40.0% and 10.0%, respectively. The OS rate of the unique RCT about inolimomab was 46.9% (33).

## Discussion

In this meta-analysis, basiliximab seemed to have the highest response rate, particularly in the gut and liver GVHD, and inolimomab treatment showed a higher infection rate. However, the survival seemed to be comparable among basiliximab, daclizumab, and inolimomab. This was the first meta-analysis comparing the efficacy and safety of different IL-2RAs, which provided valuable information for the treatment of SR-aGVHD.

Activation of T lymphocytes mediates one of the major pathophysiological mechanisms of aGVHD, which exclusively expresses the IL-2R alpha chain (46). IL-2RAs prohibit T-cell proliferation (47); however, in *in vitro* experiments, the ability of inhibiting T lymphocytes varied among different IL-2RAs. Kircher et al. (48) separated peripheral blood mononuclear cells from heparinized peripheral blood of healthy volunteers and then incubated them with 100 μg/Ml anti-CD3 monoclonal antibody. They set the level of proliferation in the absence of the compounds as 100%. At the concentrations of 0.001, 0.01, and 0.1 μg/Ml, basiliximab seemed to be stronger in terms of suppressing T-cell proliferation compared with daclizumab. Particularly, at the concentration of 0.1 μg/Ml, basiliximab could reduce T-cell proliferation from 100% to 41%, while daclizumab could reduce it only from 100% to 69%. However, at higher concentrations (e.g., 1 and 10 μg/Ml), both of them inhibited T-cell proliferation to a similar degree. Thus, T cells were more sensitive to inhibition by basiliximab in this study.

Similarly, Baan et al. (49) identified the inhibitory effect of different IL-2RAs (basiliximab, daclizumab, and inolimomab) on T-cell proliferation induced by IL-2, IL-7, and IL-15. At lower concentrations (0.1, 0.5, and 1.0 μg/Ml), basiliximab occupied the dominant position for suppressing T-cell proliferation induced by IL-2, followed by daclizumab and inolimomab ranking the last. For suppressing T-cell proliferation induced by IL-7, daclizumab seemed to be stronger than basiliximab at concentrations of 0.1 and 0.5 μg/Ml, while basiliximab seemed to be better at other concentrations (1.0, 5.0, and 10.0 μg/Ml). Irrespective of the concentration, T cells appeared to be minimally inhibited by inolimomab. In IL-15-driven T-cell proliferation, daclizumab performed better than basiliximab at concentrations higher than 0.5 μg/Ml, while the function of inolimomab was still the weakest. These results might also partly explain the fact that inolimomab was less effective than basiliximab and daclizumab in SR-aGVHD treatment. Among these three cytokines (i.e., IL-2, IL-7 and IL-15), IL-2 showed the preferential protective effects on T cells against glucocorticoid-induced apoptosis (50). Therefore, basiliximab exhibited the best efficacy in treating SR-aGVHD.

From the perspective of structure, inolimomab was a murine anti-human monoclonal antibody (7), basiliximab was a murine chimeric monoclonal antibody (8), and daclizumab was a humanized monoclonal antibody (9). The human immune system can produce its own antibodies to clear rodent antibodies rapidly because they are foreign proteins, leading to reduced efficacy (51, 52). An increased risk of an infusion reaction may exist as well (53). Moreover, a longer half-life may help to inhibit T cells more effectively. The half-lives of basiliximab and daclizumab are 7 days and 21–25 days (8, 9), respectively, while the half-life of inolimomab is only 44.5 h (7). It might partially explain the higher ORRs of basiliximab (81%) and daclizumab (71%), while that of inolimomab was only 50%.

It is reported that IL-2RA may suppress regulatory T cells (54) which possibly leads to chronic GVHD after treatment (55, 56). However, aGVHD was a significant risk factor of cGVHD, and more than 65% of patients with grades II to IV aGVHD would develop cGVHD (57–59). This was similar to the rate of cGVHD in the present study. Thus, it was suggested that the suppression of regulatory T cells caused by IL-2RA might have an influence on cGVHD, however, the incidence of cGVHD could also be driven by the prior SR-aGVHD, which needs to be further explored by prospective RCTs.

In this study, basiliximab, daclizumab, inolimomab, and denileukin diftitox were found to have similar efficacy in aGVHD of the skin, while basiliximab seemed to be better in aGVHD of the gut and liver. Clinically, aGVHD of the gut and liver significantly increased the risk of transplant-related mortality (60). Therefore, basiliximab could improve the prognosis to a much greater extent, especially in severe SR-aGVHD.

This study had several limitations. First, the generalizability of this meta-analysis was limited by various circumstances due to the heterogeneity originating from different study designs and the process of conducting and analyzing, which might influence the accuracy of our results. Second, the time frame for the evaluation of the response rate at 1 month after IL-2RA treatment was prolonged because different studies had different time points. That is, the earliest studies evaluating at 3 weeks while the latest studies evaluating at 6 weeks after treatment with IL-2RAs were enrolled in this analysis, which might have influenced the study outcomes. Third, one more drawback was the lack of prospective RCTs to compare distinct categories of IL-2RA directly. Instead, most studies that could be used for analysis were retrospective studies with relatively limited sample sizes. Finally, several biases might have been introduced into this meta-analysis, including unbalanced medical resources from different time points or areas. Thus, the superiority of basiliximab over other IL-2RAs in patients with SR-aGVHD needs to be validated by further studies.

## Conclusion

In conclusion, the efficacy and safety of different IL-2RAs varied. The response rate of basiliximab seemed to be the highest, followed by daclizumab. More prospective RCTs are needed to compare the efficacy and safety of different IL-2RAs.

## Data Availability Statement

The raw data supporting the conclusions of this article will be made available by the authors, without undue reservation.

## Author Contributions

Two reviewers J-XL and M-ZS conducted the study selection and data extraction independently. A third reviewer X-DM arbitrated when the former two reviewers had diverse opinions X-DM and X-JH designed the study. J-XL, M-ZS, L-PX, X-HZ, YW, and K-YL collected the data. J-XL, M-ZS, and S-DH analyzed the data and drafted the manuscript. All authors contributed to the article and approved the submitted version.

## Funding

This work was supported by the National Key Research and Development Program of China (grant number 2017YFA0104500), CAMS Innovation Fund for Medical Sciences (CIFMS) (grant number 2019-I2M-5-034), the Foundation for Innovative Research Groups of the National Natural Science Foundation of China (grant number 81621001), the Key Program of the National Natural Science Foundation of China (grant number 81930004), and the Fundamental Research Funds for the Central Universities.

## Conflict of Interest

The authors declare that the research was conducted in the absence of any commercial or financial relationships that could be construed as a potential conflict of interest.

## Publisher’s Note

All claims expressed in this article are solely those of the authors and do not necessarily represent those of their affiliated organizations, or those of the publisher, the editors and the reviewers. Any product that may be evaluated in this article, or claim that may be made by its manufacturer, is not guaranteed or endorsed by the publisher.
