# Supplementary material for: Meta-Analysis of Interleukin-2 Receptor Antagonists as the Treatment for Steroid-Refractory Acute Graft-Versus-Host Disease
Source: Front Immunol. 2021 Sep 21;12:749266. doi: 10.3389/fimmu.2021.749266 (PMC8490710; doi:10.3389/fimmu.2021.749266)
Supplement: Supplementary file 1 [file Table_1.docx]

**Supplementary Table 1. The underlying disease of patients in 31 included studies**

| **Studies** | **Disease (*n*)** | | | |
| --- | --- | --- | --- | --- |
|  | **AL** | **MDS/MPN** | **AA/PNH** | **Others** |
| **Basiliximab-based treatment** |  |  |  |  |
| Liu, 2020 | 162 | 32 | 19 | 17 |
| Tang, 2020 | 69 | 9 | 16 | 6 |
| Tan, 2017 | 55 | 2 | 4 | 4 |
| Chakupurakal, 2015 | 11 | 0 | 0 | 3 |
| Nadeau, 2016 | 10 | 2 | 2 | 7 |
| Jaiswal, 2016 | 5 | 0 | 4 | 1 |
| Wang, 2011 | NA | NA | NA | NA |
| Schmidt-Hieber, 2005 | 11 | 0 | 0 | 12 |
| Massenkeil, 2002 | 7 | 1 | 0 | 9 |
| **Daclizumab-based treatment** |  |  |  |  |
| Tao, 2015 | 46 | 8 | 0 | 10 |
| Rager, 2011 | 9 | 0 | 0 | 8 |
| Rao, 2009 | 2 | 2 | 1 | 17 |
| Miano, 2009 | 6 | 0 | 0 | 7 |
| Hui, 2008 | 9 | 1 | 0 | 2 |
| Perales, 2007 | 53 | 0 | 1 | 3 |
| Teachey, 2006 | NA | NA | NA | NA |
| Bordigoni, 2006 | NA | NA | NA | NA |
| Wolff, 2005 | 10 | 1 | 1 | 9 |
| Srinivansan, 2004 | 0 | 0 | 1 | 3 |
| Willenbacher, 2001 | 6 | 1 | 0 | 5 |
| Preziprka, 2000 | 32 | | 1 | 10 |
| **Inolimomab-based treatment** |  |  |  |  |
| Girerd, 2017 | 21 | 4 | 0 | 24 |
| Garcia-Cadenas, 2017 | 34 | 18 | 0 | 46 |
| Groningen, 2016 | NA | NA | NA | NA |
| Girerd, 2013 | 15 | 3 | 1 | 14 |
| Garcia-Cadenas, 2013 | 29 | 29 | 0 | 34 |
| Xhaard, 2011 | 12 | 0 | 0 | 8 |
| Pinana, 2006 | 8 | 1 | 2 | 29 |
| Bay, 2005 | 33 | 0 | 4 | 48 |
| **Denileukin diftitox treatment** |  |  |  |  |
| Shaughnessy, 2005 | 6 | | 1 | 15 |
| Ho, 2004 | 15 | | 0 | 15 |

NA, not available; AL, acute leukemia; MDS, myelodysplastic syndrome; MPN, myeloproliferative neoplasm; AA, aplastic anemia; PNH, paroxysmal nocturnal hemoglobinuria.
