# Supplementary figures and images for: Meta-Analysis of Interleukin-2 Receptor Antagonists as the Treatment for Steroid-Refractory Acute Graft-Versus-Host Disease
Source: Front Immunol. 2021 Sep 21;12:749266. doi: 10.3389/fimmu.2021.749266 (PMC8490710; doi:10.3389/fimmu.2021.749266)

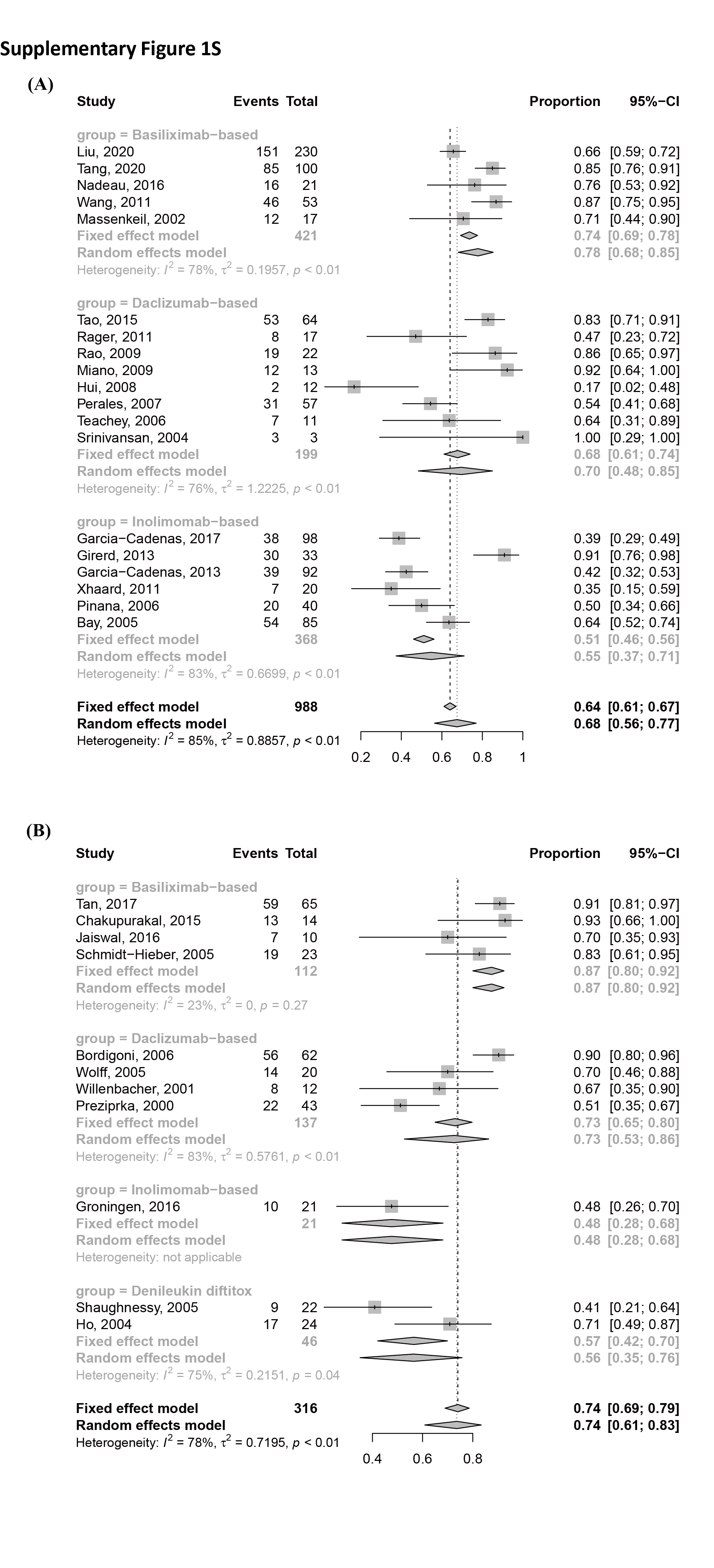

Supplement: Supplementary file 2 [file Image_1.jpeg]

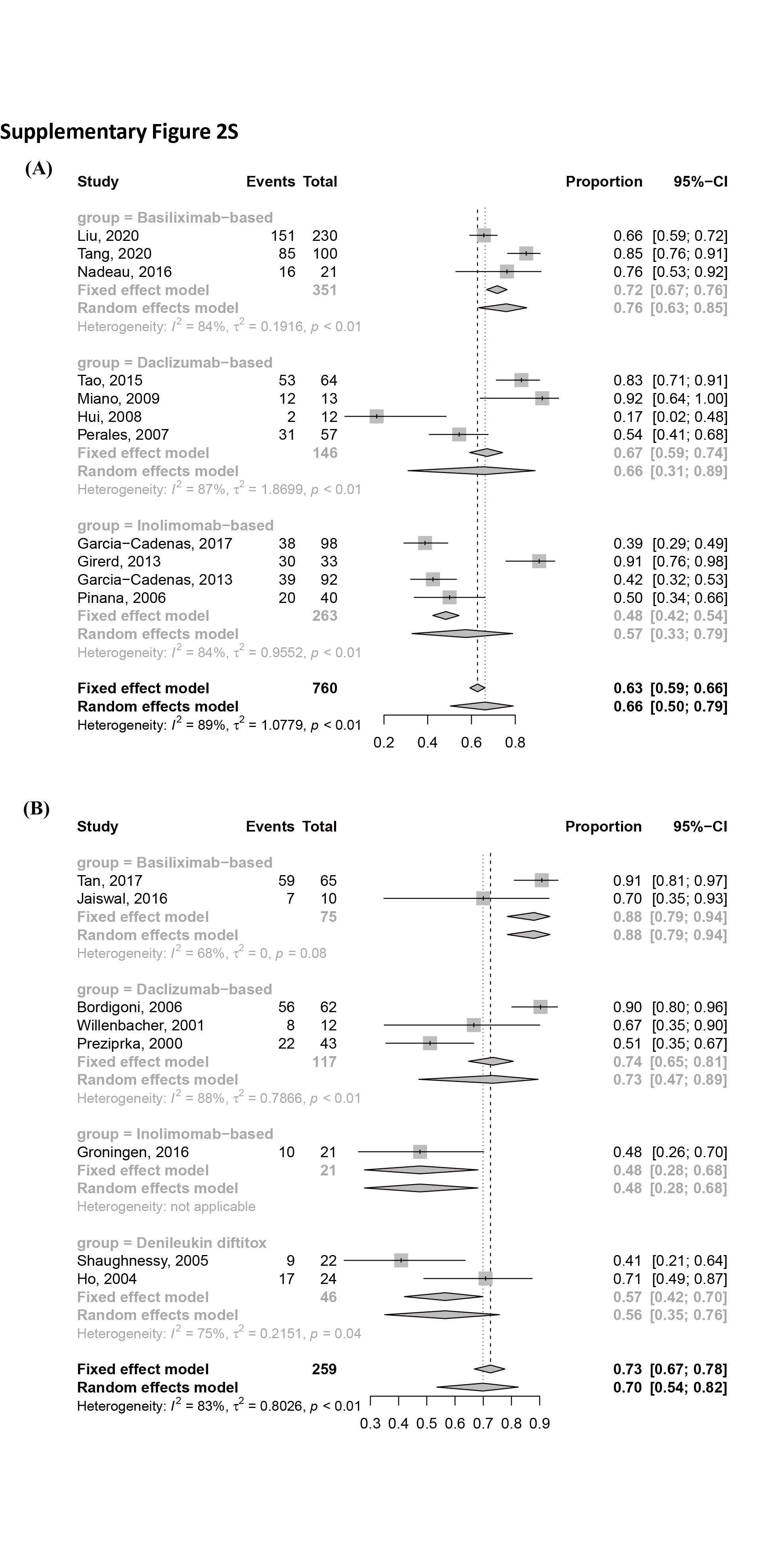

Supplement: Supplementary file 3 [file Image_2.jpeg]

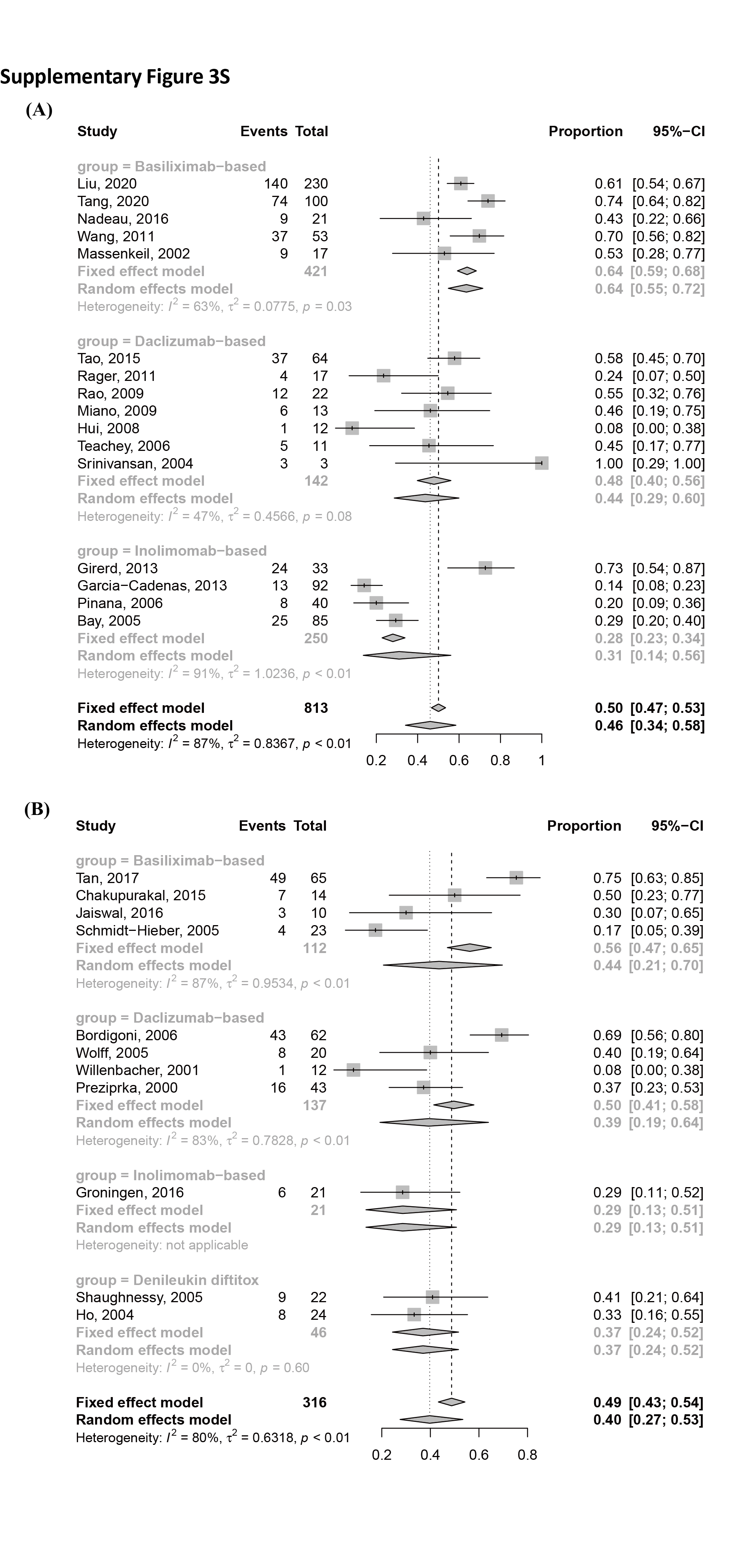

Supplement: Supplementary file 4 [file Image_3.jpeg]

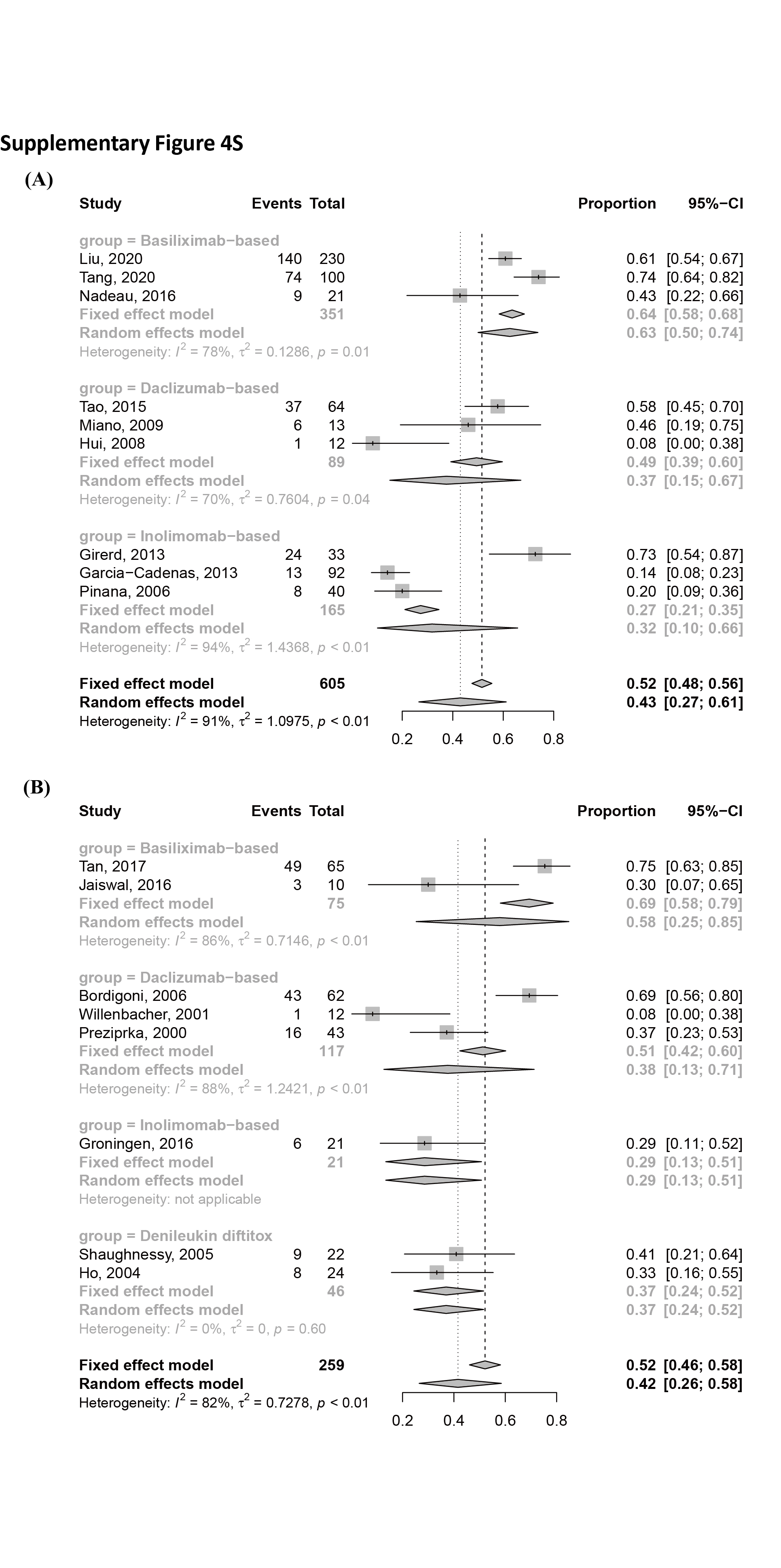

Supplement: Supplementary file 5 [file Image_4.jpeg]

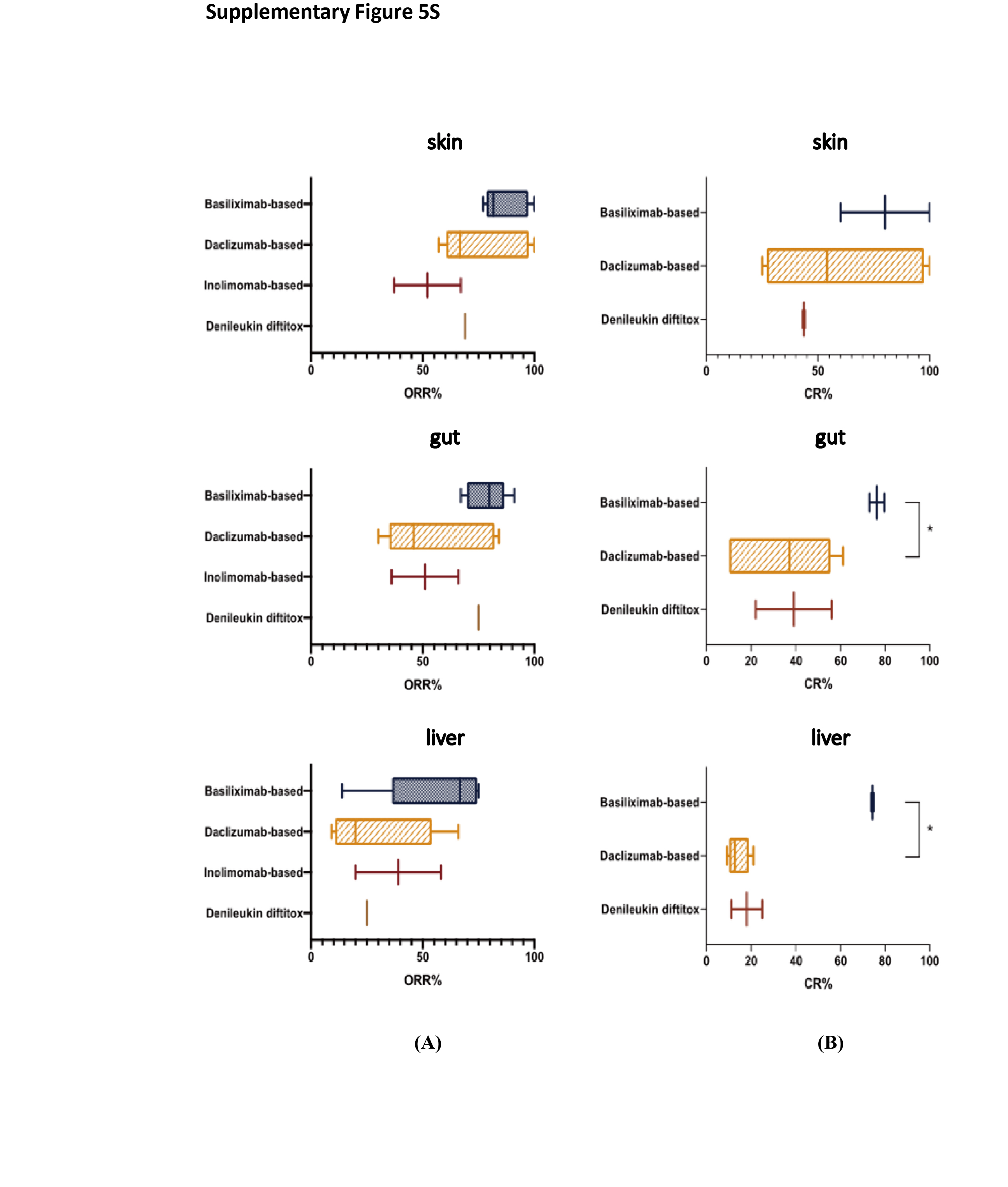

Supplement: Supplementary file 6 [file Image_5.jpeg]

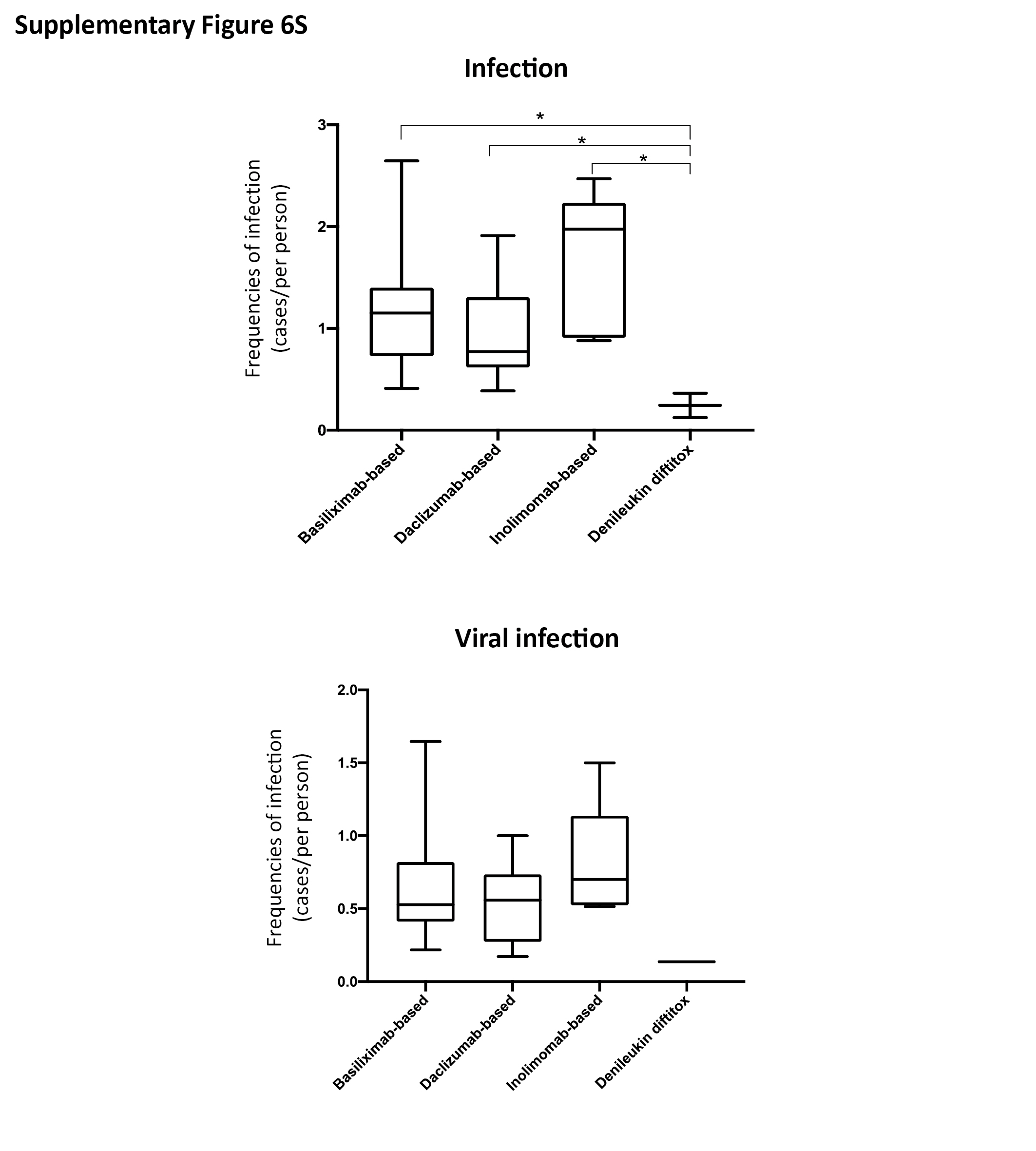

Supplement: Supplementary file 7 [file Image_6.jpeg]
